# Advanced Feature Extraction and Selection Approach Using Deep Learning and Aquila Optimizer for IoT Intrusion Detection System

**DOI:** 10.3390/s22010140

**Published:** 2021-12-26

**Authors:** Abdulaziz Fatani, Abdelghani Dahou, Mohammed A. A. Al-qaness, Songfeng Lu, Mohamed Abd Elaziz

**Affiliations:** 1 School of Computer Science and Technology, Huazhong University of Science and Technology, Wuhan 430074, China; aafatani@uqu.edu.sa; 2 Computer Science Department, Umm Al-Qura University, Makkah 24381, Saudi Arabia; 3 LDDI Laboratory, Faculty of Science and Technology, University of Ahmed DRAIA, Adrar 01000, Algeria; dahou.abdghani@univ-adrar.edu.dz; 4 Faculty of Engineering, Sana’a University, Sana’a 12544, Yemen; 5 State Key Laboratory for Information Engineering in Surveying, Mapping and Remote Sensing, Wuhan University, Wuhan 430079, China; 6 School of Cyber Science & Engineering, Huazhong University of Science and Technology, Wuhan 430074, China; 7 Shenzhen Huazhong University of Science and Technology Research Institute, Shenzhen 518057, China; 8 Department of Mathematics, Faculty of Science, Zagazig University, Zagazig 44519, Egypt; abd_el_aziz_m@yahoo.com; 9 Artificial Intelligence Research Center (AIRC), Ajman University, Ajman 346, United Arab Emirates; 10 Faculty of Computer Science & Engineering, Galala University, Suze 435611, Egypt

**Keywords:** feature selection, cybersecurity, sustainable computing, intrusion detection system, Aquila optimizer, swarm Intelligence, internet of things (IoT)

## Abstract

Developing cyber security is very necessary and has attracted considerable attention from academy and industry organizations worldwide. It is also very necessary to provide sustainable computing for the the Internet of Things (IoT). Machine learning techniques play a vital role in the cybersecurity of the IoT for intrusion detection and malicious identification. Thus, in this study, we develop new feature extraction and selection methods and for the IDS system using the advantages of the swarm intelligence (SI) algorithms. We design a feature extraction mechanism depending on the conventional neural networks (CNN). After that, we present an alternative feature selection (FS) approach using the recently developed SI algorithm, Aquila optimizer (AQU). Moreover, to assess the quality of the developed IDS approach, four well-known public datasets, CIC2017, NSL-KDD, BoT-IoT, and KDD99, were used. We also considered extensive comparisons to other optimization methods to verify the competitive performance of the developed method. The results show the high performance of the developed approach using different evaluation indicators.

## 1. Introduction

Internet applications help people and society in many fields, including teaching, electronic commerce (EC), electronic learning, entertainment, electronic communication, and others [1]. Along with these applications, cybersecurity issues have been raised due to the vulnerability of the internet applications due to the wide expansion of the networks and the massive emergence of malicious intrusion [1]. Therefore, building security systems is very necessary, and many industrial and academic organizations have developed different systems and solutions. Intrusion detection systems (IDS) are very important for the cybersecurity of the internet of things (IoT) architecture, including also cloud and fog computing.

Previously, different methods have been developed for intrusion detection systems (IDS) using traditional machine learning methods, such as k-means clustering [2,3], decision tree (DT) [4,5], k-nearest neighbor (kNN) [6,7], support vector machine (SVM) [8,9], and other traditional machine learning (ML) approaches. With the wide spread of the deep learning methods, in recent years thy are also adopted for IDS, such as multi-layered perceptron neural network [10], convolutional neural networks (CNN) [11], and deep recurrent neural network (RNN) [12]. However, deep leaning approaches required big size of features to achieve high classification accuracy rates.

Feature selection (FS) is a necessary preprocessing step in ML applications [13]. In literature, there are different approaches proposed for IDS by improving new FS methods that boosted the efficiency of the IDS. For example, grey wolf optimizer (GWO) [14,15], crow search algorithm (CSA) [16], genetic algorithm (GA) [17,18,19], whale optimization algorithm [20], random harmony search (RHS) [21], and also the well-known, particle swarm optimization (PSO) [22]. Although these approaches showed significant performance, they suffer from certain limitations. For instance, some of them may be stuck at local optima, which degrades the convergence rate and finally on the quality of find decision.

In the current study, we present an alternative FS approach for IDS using a recently proposed optimization algorithm called Aquila optimizer (AQU). The AQU was developed by Abualigah et al. [23], which mimics the behaviors of Aquila in nature. It was assessed with different engineering and optimization problems, and it illustrated competitive performance compared to traditional optimization algorithms. The AQU also received wide attention, as it was adopted to solve different problems, such as industrial engineering optimization problems [24], medical image processing [25], and others [26]. The traditional AQU suffers from slow convergence; thus, we use the binary version to boost its performance.

In this study, we first apply a light feature extraction approach based on CNN to obtain features from the used datasets. Thereafter, the developed AQU algorithm is utilized to select a subset of the optimal features that reflect the characteristics of the datasets. We use four public benchmark datasets including BoT-IoT, NSL-KDD, CIC2017, and KDD99, to evaluate the developed approach, which showed significant performance. In short, the contribution presented in this paper can be summarized as follows:

1. Using the combination of deep learning and Aquila optimizer (AQU) to enhance IoT security.
2. A feature extractor technique based on CNN is applied to extract relevant features from the datasets,
3. A binary version of the Aquila optimizer is adopted as an FS technique that is used to select optimal features and enhance the classification accuracy.
4. Extensive evaluation is carried out with four public datasets and extensive comparisons to other methods to confirm the quality of the developed approach.

The remaining parts of this paper are presented as: Section 2 summarizes several related studies presented in recent years. The basics of the used methods are described in Section 3, whereas the presented IoT approach is introduced in Section 4. Moreover, the evaluation experiments and results outcomes are described in Section 5. Section 6 presents the conclusion and future work.

## 2. Related Works

In this section, we summarize a number of previous approaches proposed for IDS in IoT and cloud. Shafiq et al. [27] presented an efficient feature selection technique for IoT malicious traffic identification using the Bot-IoT dataset. They used the objective soft set for feature extraction, and they developed a new feature selection method called, CorrACC. Haddadpajouh et al. [28] applied gray wolves optimization (GWO) to improve the multi-kernel SVM for IoT cloud-edge gateway malware detection. GWO is utilized as an FS method which enhanced the classification accuracy. It was evaluated and compared to previous methods, and it reached good results. A  wrapper-based FS method called, CorrAUC was developed by [29] for malicious traffic detection for IoT environments, using Bot-IoT datasets. This method was tested with four machine learning algorithms, and it showed significant performance in reducing feature seize and boosting classification accuracy. Davahli et al. [30] presented a hybrid FS technique using GWO and GA algorithms. This method was employed with the SVM classifier to detect anomalies in wireless sensor networks (WSNs). Mafarja et al. [31] developed a new wrapper feature selection method using an augmented Whale Optimization Algorithm (WOA) for IoT attacks identification. The augmented WOA was employed to handle the high dimensionality of the datasets and to enhance the classification accuracy. They used two transfer functions, S-shaped and V-shaped, into the WOA to boost its performance. The enhanced WOA showed better performance compared to the traditional WOA. Sekhar et al. [32] developed an IDS approach based on Fruitfly optimization with deep Autoencoder. They used fuzzy C-Means rough parameters for data processing to deal with the missing data from the used datasets. After that, the robust features can be extracted from Autoencoder with multi-hidden layers. Then, the extracted features are fed to the BPN (Back Propagation Neural Network) for attacks classification. The Fruitfly optimization algorithm is used to optimize the neurons in the Deep Autoencoder hidden layers. This method was evaluated with UNSW-NB15 and NSL-KDD datasets, and it showed competitive performance. Dwivedi [33] presented an alternative FS approach depending on the grasshopper optimization algorithm (GOA) for IDS. The main goal of this approach is to integrate GOA with the integration of ensemble feature selection (EFS) and creating a new method called EFSGOA. The EFS is used to rank the features to select the relevant features, and then the GOA is used for identifying the significant features. This approach was tested with KDD Cup 99 and NSL-KDD datasets, and it obtained high accuracy rates. Kan et al. [34] used the adaptive PSO and CNN for IDS in the IoT network. In this method, APSO-CNN is working by optimizing one-dimensional CNN structure parameters using the PSO algorithm. It was tested with comparison to other CNN-based methods, and the outcomes showed that the application of PSO has a significant impact on the performance of the CNN. The PSO was also adopted in other IDS systems, such as [35,36,37,38].

## 3. Background

### Aquila Optimizer (AQU)

This section introduces the basic formulation of the Aquila Optimizer (AQU) [23]. In general, the AQU algorithm mimics Aquila’s social behavior in order to catch its prey. AQU is a population-based optimization technique, similar to other metaheuristic (MH) techniques, that begins by forming an initial population *X* with *N* agents. The following equation was used to carry out this procedure.

(1) $$X_{ij}=r_{1}\times \left(UB_{j}-LB_{j}\right)+LB_{j},\;\;\;i=1,2,.....,N\;j=1,2,\ldots ,Dim$$

In Equation (1), $UB_{j}$ and $LB_{j}$ represent limits of the search space. $r_{1}\in \left[0,1\right]$ denotes a random value and Dim is the dimension of agent.

The AQU technique’s next step is to do either exploration or exploitation until the best solution is found. There are two ways for exploration and exploitation, according to [23].

The best agent $X_{b}$ and the average of agents ($X_{M}$) are employed in the exploration, and its mathematical formulation is given as:

(2) $$X_{i}\left(t+1\right)=X_{b}\left(t\right)\times \left(\frac{1-t}{T}\right)+\left(X_{M}\left(t\right)-X_{b}\left(t\right)*rand\right),$$

(3) $$X_{M}\left(t\right)=\frac{1}{N}\sum_{i=1}^{N}X\left(t\right),\;\forall j=1,2,\ldots ,Dim$$

The search during the exploration phase is controlled by $\left(\frac{1-t}{T}\right)$ in Equation (2). The maximum number of generations is denoted by *T*.

The exploration phase employs the Levy flight (Levy(D) distribution and $X_{b}$ to update the solutions, and this is represented as:

(4) $$X_{i}\left(t+1\right)=X_{b}\left(t\right)\times Levy\left(D\right)+X_{R}\left(t\right)+\left(y-x\right)*rand,$$

(5) $$Levy\left(D\right)=s\times \frac{u\times \sigma }{{\left|\upsilon \right|}^{\frac{1}{\beta }}},\;\sigma =\left(\frac{\Gamma \left(1+\beta \right)\times sine\left(\frac{\pi \beta }{2}\right)}{\Gamma \left(\frac{1+\beta }{2}\right)\times \beta \times 2^{\left(\frac{\beta -1}{2}\right)}}\right)$$

In Equation (5), s=0.01 and β=1.5. *u* and υ denotes the random values. $X_{R}$ stands for randomly chosen agent. In addition, *y* and *x* stands for two parameters used to simulate the spiral shape:

(6) y=r×cos(θ),x=r×sin(θ)

(7) $$r=r_{1}+U\times D_{1},\;\theta =-\omega \times D_{1}+\theta_{1},\;\theta 1=\frac{3\times \pi }{2}$$

In Equation (7), ω=0.005 and U=0.00565. $r_{1}\in \left[0,20\right]$ refers to a random value.

The first technique used in [23] to enhance the agents in the exploitation phase depends on $X_{b}$ and $X_{M}$, similar to exploration, and it is formulated as:

(8) $$X_{i}\left(t+1\right)=\left(X_{b}\left(t\right)-X_{M}\left(t\right)\right)\times \alpha -rnd+\left(UB\times rnd+LB\right)\times \delta$$

In Equation (8), UB=(UB−LB), α and δ stands for the exploitation adjustment parameters. rnd∈[0,1] is random value.

The agent can be updated using $X_{b}$, Levy, and the quality function QF in the second exploitation strategy. This strategy’s mathematical definition is as follows:

(9) $$X_{i}\left(t+1\right)=QF\times X_{b}\left(t\right)-GX-G_{2}\times Levy\left(D\right)+rnd\times G_{1}$$

$$GX=(G_{1}\times X\left(t\right)\times rnd)$$

(10) $$QF\left(t\right)=t^{\frac{2\times rnd()-1}{{\left(1-T\right)}^{2}}}$$

In addition, $G_{1}$ stands for the motions used to track the optimal individual solution, as seen in the following equation:

(11) $$G_{1}=2\times rnd\left(\right)-1,\;G_{2}=2\times \left(1-\frac{t}{T}\right)$$

In Equation (11), rnd is a random value. Moreover, $G_{2}$ stands for parameter which decreasing from 2 to 0, and it is updated as:

(12) $$G_{2}=2\times \left(1-\frac{t}{T}\right)$$

## 4. Proposed Model

**Figure 1:**
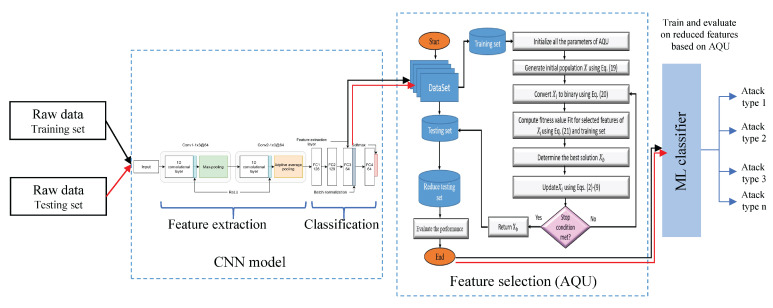
Structure of presented IoT security model.

Figure 1 depicts the structure of an IDS security scheme for IoT systems. The suggested system is divided into two phases: a feature extraction phase using an efficient CNN based method and a feature selection phase based on the developed AQU algorithm. The presented AQU is based on improving the behavior of classical AQU to make it suitable for the FS problem by implementing its binary version. In the following sections, a description of each stage of the developed IoT security model is given.

### 4.1. Representation of Collect IoT Dataset

The fundamental representation of IoT traffic data that will be employed as input to the next stage of the proposed approach is presented in this section. Consider TS, which is a sample of IoT traffic and is written as:

(13) $$\begin{array}{c} TS=\left[\begin{array}{cccc} tf_{11} & tf_{12} & ... & tf_{1d}\\ tf_{21} & tf_{22} & ... & tf_{2d}\\ ... & ... & ... & ...\\ tf_{n1} & tf_{n2} & ... & tf_{nd} \end{array}\right] \end{array}$$

In Equation (15), $TS_{i}$ denotes the *i*th set of features of traffic (i.e., $\left[tf_{11},tf_{12}m\ldots ,tf_{1d}\right]$). *d* and *n* are the number of features and samples respectively. Thereafter, the dataset is normalized based on the *min* − *max* approach that defined:

(14) $$\begin{array}{c} DN_{ij}=\frac{tf_{ij}-min\left(TS_{j}\right)}{max\left(TS_{j}\right)-min\left(TS_{j}\right)} \end{array}$$

where $tf_{ij}$ stands for the *j*th feature of sample *i*.

Therefore, the normalization of TS is formulated as:

(15) $$\begin{array}{c} NTS=\left[\begin{array}{cccc} DN_{11} & DN_{12} & ... & DN_{1d}\\ DN_{21} & DN_{22} & ... & DN_{2d}\\ ... & ... & ... & ...\\ DN_{n1} & DN_{n2} & ... & DN_{nd} \end{array}\right] \end{array}$$

The next step is to extract the feature using DL model from NTS. The following process of extracting the feature using DL is given in the following section.

### 4.2. Convolutional Neural Network for Feature Extraction

Convolutional neural networks are well-known deep learning (DL) models applied to solve different problems in image classification, text classification, speech recognition, and object detection. CNN’s are commonly used in computer vision problems. However, CNN’s can be extended and employed in research fields tackling natural language processing [39,40,41], image processing [42,43], green computing [44,45], remote sensing [46,47], and others [48]. Unlike traditional machine learning algorithms that rely on handcrafted feature extraction, CNNs can automatically learn and represent complex features. Meanwhile, CNN’s based models can vary in terms of the type and number of convolution layers, kernel size and its initialization technique, pooling operation, and the fully connected layers.

**Figure 2:**
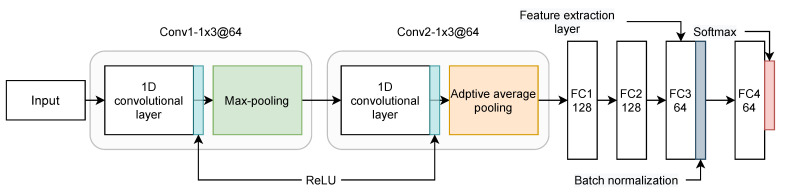
The feature extraction module based on a proposed CNN architecture.

At this stage, the main objective is to learn meaningful representations from the raw data, which helps maximize the overall framework’s recognition accuracy. After the learning phase using the CNN model, the feature selection algorithm is used to filter the extracted features by selecting the most important features only that maximize the classification accuracy. The CNNs are characterized by a core ability that shares weights between multiple layers to minimize the model complexity [49]. The proposed CNN architecture is illustrated in Figure 2, and it is composed of the following layers: (2) Convolutional layers (Conv), (2) Pooling layers, and (4) Fully connected layers (FC). The full network can be summarized as (Conv1−1×3@64)→(Conv2−1×3@64)→(FC1−128)→(FC2−128)→(FC3−64)→(BN−64)→(FC4−64) where: (1) Conv1 is the first convolutional layer with 64 filters, kernel of size 3, stride of size 1. Conv1 uses the rectified linear unit (ReLU) [50] as a non-linear function followed by a dropout regularization with a rate equal to 0.5 and a max-pooling operation of size 2, (2) Conv2 is the second convolutional layer similar to Conv1 with the only difference is the usage of an adaptive average pooling layer [51] instead of max-pooling, (3) FC1, FC2, and FC3 are fully connected layer having 128, 128 and 64 neurons, respectively. FC1, FC2, and FC3 are used as feature extraction layers to output the learned features from the raw input, (4) BN stands for batch normalization operation, and (4) FC4 is the final FC layer to output the classification predictions.

The network uses a 1D convolution operation in each convolution layer to learn the raw data activation maps after applying a fixed kernel of size 1×3 and then uses a max-pooling operation to extract the most relevant features. The convolution operation can be represented as:

(16) $$X_{j}^{l}=\sum_{i\in M_{j}}x_{j}^{l-1}k_{ij}^{l}+b_{j}^{l}$$

where $x_{j}^{l-1}$ is the output activation map of the previous layer l−1. $k_{ij}^{l}$ represents the kernel weights while $b_{j}^{l}$ represents the bias value.

To learn complex feature representations from the input data, a non-linear function is applied in the convolution operation, which can be defined as in the following equation:

(17) $$x_{j}^{l}=ReLU\left(X_{j}^{l}\right)$$

where the *l* and *j* stands for the *l* layer and the *j* channel, respectively. The $x_{j}^{l}$ is the activation map extracted from the *l* layer. The ReLU function is introduced in Equation (18).

(18) ReLU(z)=max(0,z)

The final feature representation of each input sample is obtained after pooling together the generated activation maps. Two types of pooling operations have been employed in this architecture to extract the most relevant features and down-sampling the features space and learning parameters which helps the model train faster.

The final output from Conv2 is fed to a series of fully connected layers where FC3 is used to extract the features (input samples embeddings). The final output from FC3 is fed to FC4 which output the classification results. FC4 applies a Softmax function to generate the probabilities of an input sample to belong to a specific class. Batch normalization (BN) and dropout regularization techniques are used to overcome the network over-fitting and improve the training speed and convergence.

### 4.3. Feature Selection

**Figure 3:**
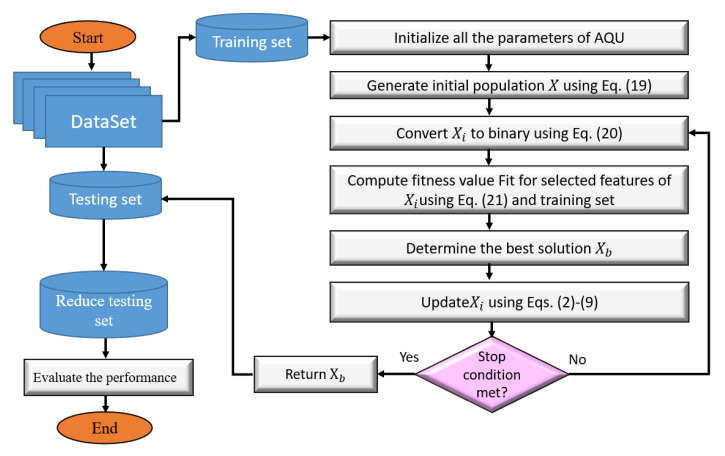
The FS approach using AQU algorithm.

The steps of the presented FS model (as in Figure 3) that are used to enhance the security in IoT environment are discussed in this section. In general, the main objective of these steps is to determine the important features that are chosen based on their quality. This is accomplished by the usage of a binary version of AQU. The presented FS approach, named AQU, begins by creating *X* initial population of *N* agents; after that, reducing the training data by selecting only the features that correspond to ones in the Boolean version of the current solution. The efficiency of the determined feature is then calculated using the KNN classifier’s error classification. Following that, the best agent with the smallest fitness value is assigned. The agents in the current population are updated based on this best agent and the AQU until they find the best solution.

#### 4.3.1. Generation Initial Population

The presented AQU begins by splitting the tested benchmark data into 80% and 20% training and testing sets, respectively. The beginning population *X* that consists of *N* solutions is formed using Equation (19).

(19) $$X_{i}=LB+rand\left(1,D\right)\times \left(UB-LB\right)$$

In Equation (19), *D* stands for the number of features. rand(1,D) represents a random vector with *D* values. LB and UB stand for the boundaries of the search space.

#### 4.3.2. Updating Population

This stage starts with Equation (20) turning $X_{i},i=1,2,\ldots ,N$ into its Boolean value $BX_{i}$.

(20) $$BX_{ij}=\left\{\begin{array}{cc} 1 & if\;X_{ij}>0.5\\ 0 & otherwise \end{array}\right.$$

Based on the output of Equation (20), the number of feature selection is reduced by ignoring the irrelevant features that corresponding zeros value in $BX_{i}$. Then the fitness value is computed using Equation (21).

(21) $$Fit_{i}=\lambda \times \gamma_{i}+\left(1-\lambda \right)\times \left(\frac{|BX_{i}|}{D}\right)$$

where λ∈[0,1] stands for the weights applied to control the balancing between the ratio of relevant features $\left(\frac{|BX_{i}|}{D}\right)$ and error of classification $\gamma_{i}$. In this study, the $\gamma_{i}$ is computed based on the KNN classifier using the training set.

Thereafter, the best Fit and its corresponding agent $X_{b}$ (i.e., the best one) are determined. Then update the current agents with operators of AQU as discussed in Section 4.

#### 4.3.3. Terminal Criteria

The stopping conditions are reviewed at this stage, and the updated stage is conducted again when these conditions are not met. Otherwise, the learning process is terminated, and $X_{b}$ using as the output that is utilized to minimize the testing set in the next stage.

#### 4.3.4. Validation Stage

To evaluate the presented AQU’s efficiency as an FS approach, the features of the testing set are reduced based on the binary of $X_{b}$. Then several performance measures based on the decreased features are employed to compute the quality of the classification process. Algorithm 1 presents the whole description of the presented IoT technique to identify the intrusion.
**Algorithm 1** Proposed FS For IoT security.1:Input: total number of generations (*T*), and  number of agents (*N*).2:Use Equation (14) to normalize the collected IoT data.3:Using proposed CNN technique to extract the features (as in Section 4.2).4:After extracting the features, divide the data into training and testing sets.5:Use Equation (19) to generate population *X*.6:Put t=1.7:**while** t<=T **do**8:   Apply Equation (20) to generate the Binary version of $X_{i}$.9:   Use Equation (21) to calculate the fitness value $Fit_{i}$ for $X_{i}$.10:   Find the best agent $X_{b}$.11:   Enhance $X_{i}$ as in Equations (2)–(9)12:   t=t+1.13:**end while**14:Remove irrelevant features from testing set that corresponding to zeros in $X_{b}$.15:Output: Consider $X_{b}$ as output and the evaluate the performance.

## 5. Experiment Results and Discussion

In this section, the quality of the developed IoT security technique is evaluated using a set of different datasets.

### 5.1. Performance Measures

In this study, we used a set of performance metrics to compute the efficiency of the developed IoT security method. These measures defined using the concept of confusion matrix (as in Table 1). These measure are given in the following.

- Average accuracy $\left(AV_{Acc}\right)$: The accuracy metric represents the rate of correct detection of the intrusion, and it is formulated as:
  (22) $$AV_{Acc}=\frac{1}{N_{r}}\sum_{k=1}^{N_{r}}Acc_{Best}^{k},\;$$
  $$Acc_{Best}=\frac{TP+TN}{TP+FN+FP+TN}$$
  in which $N_{r}=30$ refers to the iteration number(number of runs).
- Average Recall $\left(AV_{Sens}\right)$: $\left(AV_{Sens}\right)$ or true positive rate (TPR), represents the percentage of predicting positive intrusion. It can be computed as:
  (23) $$AV_{Sens}=\frac{1}{N_{r}}\sum_{k=1}^{N_{r}}Sens_{Best}^{k},\;Sens_{Best}=\frac{TP}{TP+FN}$$
- Average Precision $\left(AV_{Prec}\right)$: this illustrates the percentage of true positive cases among all the the positive cases. The $\left(AV_{Prec}\right)$ can be calculated as:
  (24) $$AV_{Prec}=\frac{1}{N_{r}}\sum_{k=1}^{N_{r}}Prec_{Best}^{k},Prec_{Best}=\frac{TP}{FP+TP}$$
- Performance Improvement Rate (PIR): This measure is applied to estimate the improvement rates obtained by the proposed technique. it can be computed as:
  (25) $$PIR=\frac{M_{AQU}-M_{Alg}}{M_{AQU}}\times 100$$
  where $M_{AQU}$ and $M_{Alg}$ refer to the value of measure (i.e., Precision, Accuracy, Recall, and F1-measure) of the proposed AQU and other algorithms, respectively.

### 5.2. Experimental Setup

In our experiments, Adam [52] optimizer is used to update the CNN model weights using a 0.005 learning rate. The CNN model was trained for 100 epochs using a 2024 batch size. Concerning the feature selection phase, we compared the proposed FS algorithm named AQU with existing MH techniques in the literature. The MH algorithms selected for comparison including Firefly algorithm (FFA) [53], particle swarm optimization (PSO) [54], whale optimization algorithm (WOA) [55], moth flame optimization (MFO) [56], traditional TSO, multiverse optimization algorithm (MVO) [57], Bat algorithm [58], and Grey wolf optimizer (GWO) [59]. Furthermore, we used the above mentioned MH algorithms with their default parameters based on the original implementation.

### 5.3. Dataset Description

In this section, we will illustrate in details the source and statistics of the datasets used to validate the proposed framework for the network intrusion detection task. We used four datasets, including KDDCup-99, and its refined version named NSL-KDD, Industrial IoT (IIoT) traffic data named BoT-IoT, and CICIDS-2017. The task is to detect network intrusions based on the extracted features using the CNN model as either intrusion, normal, or the attack type. The datasets are described in the following paragraphs.

**Figure 4:**
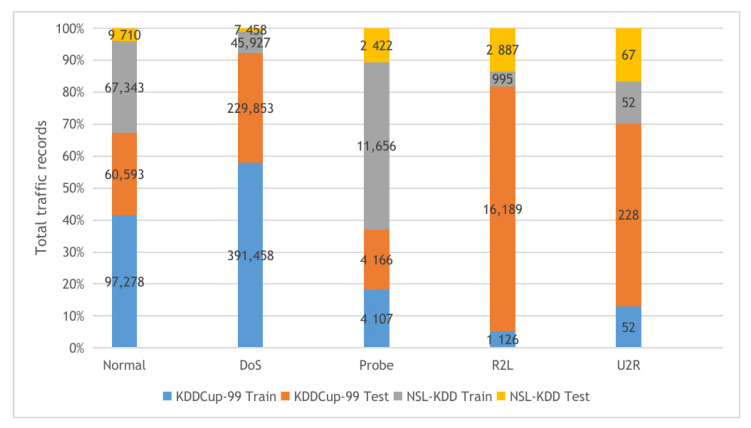
The KDDCup-99 and NSL-KDD datasets training and testing sets distribution.

**Figure 5:**
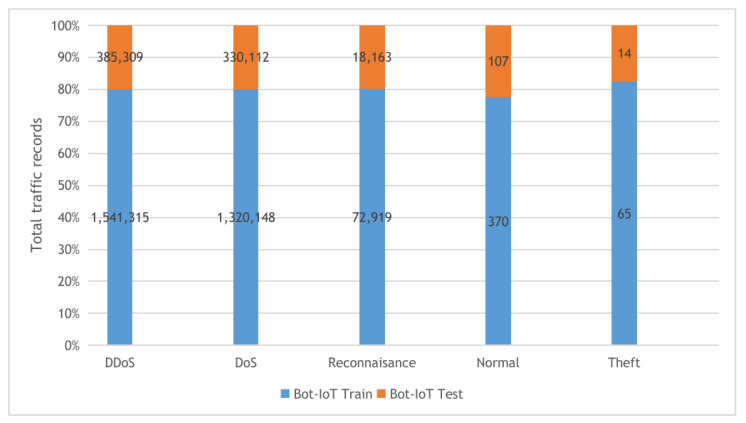
The Bot-IoT dataset training and testing sets distribution.

**Figure 6:**
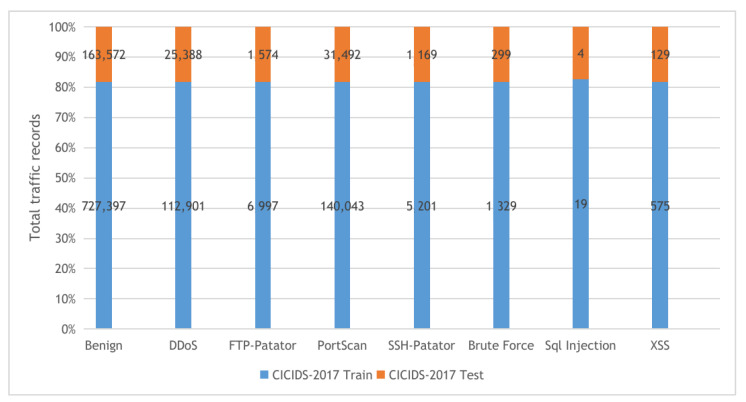
The CICIDS-2017 dataset training and testing sets distribution.

1. **KDDCup-99 and NSL-KDD**: The two datasets are described in Figure 4 with their detailed statistics. The first dataset is KDDCup-99, collected from the DARPA intrusion detection challenge (1998), incorporating 100’s users after monitoring the network traffic on 1000’s machines using UNIX operating system. The challenge period lasts for ten weeks by the MIT Lincon laboratory to store the collected traffic data in TCP dump format. Our experiments used 10% of the collected traffic data to build the KDDCup-99 dataset, which contains five attack types and 41 features. The KDDCup-99 dataset features are classified into three categories, including basic, content, and time-based traffic features. The second dataset is NSL-KDD, a derived copy from the full KDDCup-99 dataset after performing deduplication of the duplicated traffic records.
2. **BoT-IoT**: the Bot-IoT dataset [60] was collected in The center of UNSW Canberra Cyber using smart home appliances in a laboratory environment (the Cyber Range Lab). The dataset contains Industrial IoT (IIoT) traffic samples collected for IIoT experiments. The smart home appliances include weather monitoring systems, thermostats, kitchen appliances, and freezers and motion-controlled lights to record the traffic data. In our experiments, we used the 5% of the full Bot-IoT dataset, which consists of 3.6 million records, where the full dataset contains over 72 million records. The 5% of the entire dataset contains the best ten features extracted from the raw data and categorized into five main classes as described in Figure 5.
3. **CICIDS-2017**: The CICIDS-2017 [61] dataset is a collection of network traffic samples collected in CIC (The Canadian Institute for Cybersecurity at the University of New Brunswick.) for the intrusion detection task. The dataset consists of more than 1.5M PCAPs data simulating traffic data transferred in real-world using the CICFlowMeter software after analyzing 25 user behaviors covering various network protocols such as HTTP and SSH protocols. The collected data were categorized into eight main attack classes as described in Figure 6. Our experiments used the following collected CSV files: Tuesday-working hours, Friday-WorkingHours-Afternoon-PortScan, Friday-WorkingHours-Afternoon-DDos, and Thursday-WorkingHours-Morning-WebAttacks.

### 5.4. Results and Discussion

The findings of the comparison between the proposed AQU and the other MH approaches are discussed in this section. The average of the employed measures for all compared algorithms are shown in Table 2 and Table 3. For the multi-classification of the BoT-IoT, as shown in Table 2, the performance of most optimization approaches is practically similar during the training period. On the other hand, AQU, delivers excellent performance metrics. Furthermore, the developed AQU has the highest accuracy, specificity, and sensitivity, as well as the best F1-measure.

**Figure 7:**
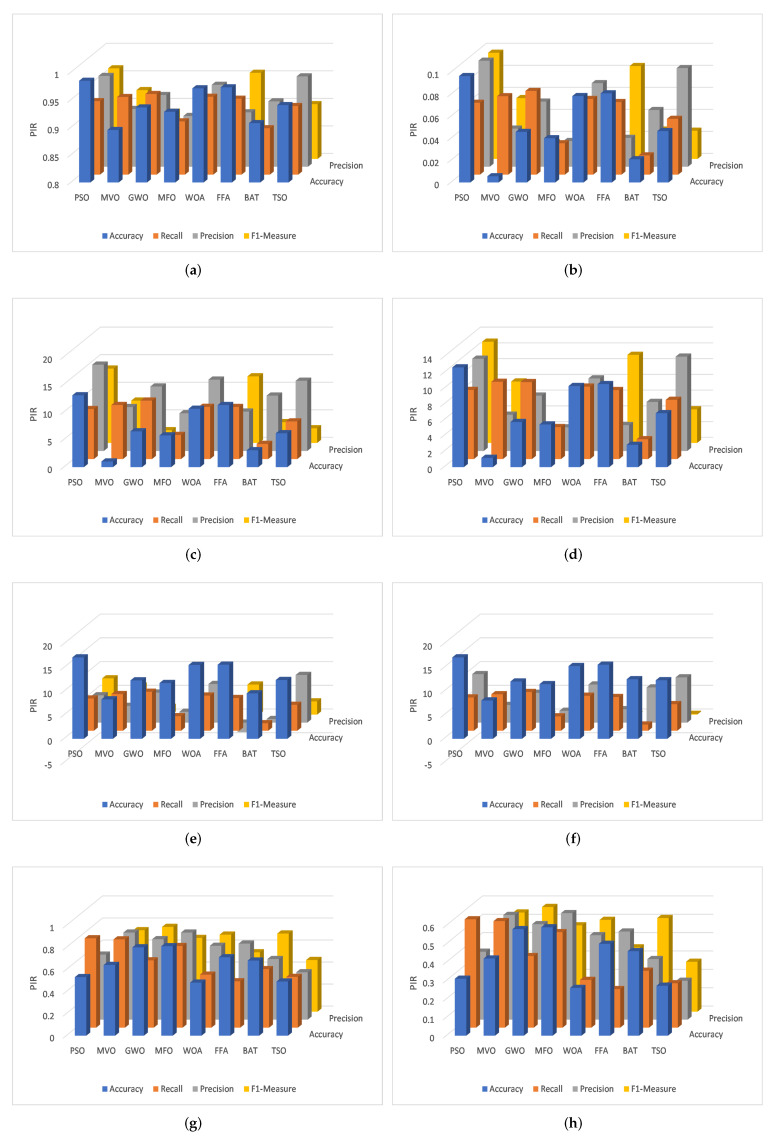
PIR for multi-classification of (**a**) Bot-IoT, (**c**) NSL-KDD, (**e**) KDDCup-99, and (**g**) CICIDS-2017 and binary classification of (**b**) Bot-IoT, (**d**) NSL-KDD, (**f**) KDDCup-99, (**h**) CICIDS-2017.

For the binary case of Bot-IoT, the AQU has better results in both the training and testing sets. Moreover, the PIR of the proposed AQU method and other optimization approaches is depicted in Figure 7a,b. For multi-classification variants, PIR ranges from 2.56 to 7.354 based on the value of accuracy, where it ranges from 1.080 to 4.410 based on the values of recall. Precision and F-measure range from 1.255 to 5.359 and 0.886 to 4.693, respectively. In binary classification case, the ranges are 2.496 to 0.0946, 0.941 to 4.210, 1.450 to 5.271, and 0.546 to 2.759, respectively.

Also, Table 2 and Figure 7c,d show the comparison results between the AQU and the compared algorithms using the NSL-KDD dataset; These results demonstrate the high performance of the proposed AQU over all compared approaches for both multi and binary classifications. As can be shown from performance measurements and the testing set results, the developed AQU behaves better in the learning phase than compared approaches. Furthermore, the developed AQU outperforms MVO with a difference of about 1.024%, and outperforms PSO with a difference of approximately 13.039%. The developed AQU outperforms existing models according to the value of recall, precision, and F-measure, with differences ranging from 2.75%, 6.85%, and 2.310% to 10.61%, 15.67%, 13.49% respectively.

**Figure 8:**
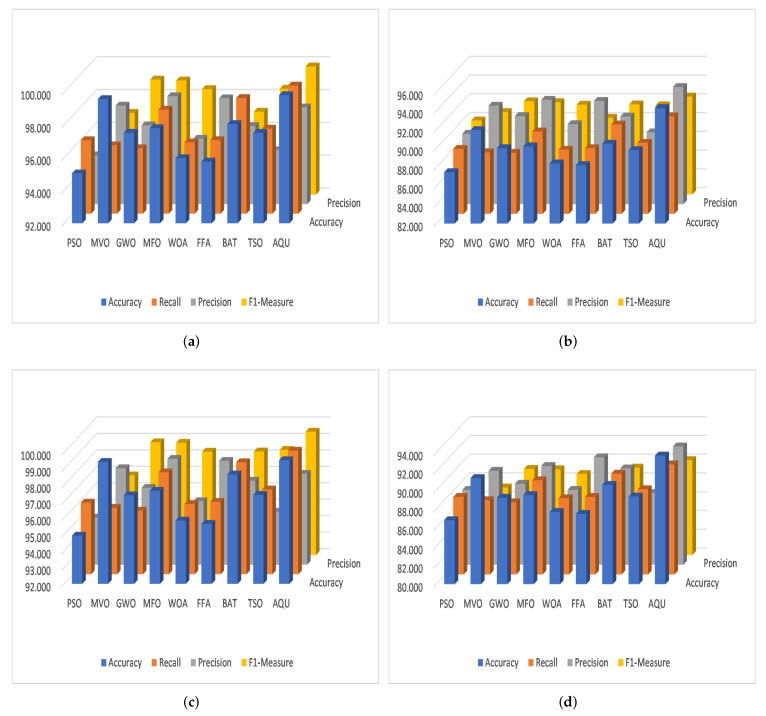
The average among the four datasets for (**a**) Training Binary, (**b**) Testing Binary, (**c**) Training Multi-classification, and (**d**) Testing Multi-classification.

For KDDCup-99, the results of the proposed AQU and all compared algorithms are shown in Table 2 (Figure 7e) and Table 3 (Figure 7f), respectively. We can see that for the multi-classification, the proposed AQU outperforms other approaches in the training stage. However, the BAT and FFA produce higher F1-measure and Precision values than other models. While AQU still outperforms MVO according to the value of accuracy, and there is only a 0.4 difference between the two. Furthermore, the advantage of AQU over binary KDDCup-99 can be seen in the comparison findings for all evaluation indicators. It achieved the best results using both training and testing datasets. Figure 8 shows the average of outcomes of all testing datasets for each algorithm. It can be seen that the AQU has a great ability to improve intrusion detection in both multi and binary classification instances.

**Figure 9:**
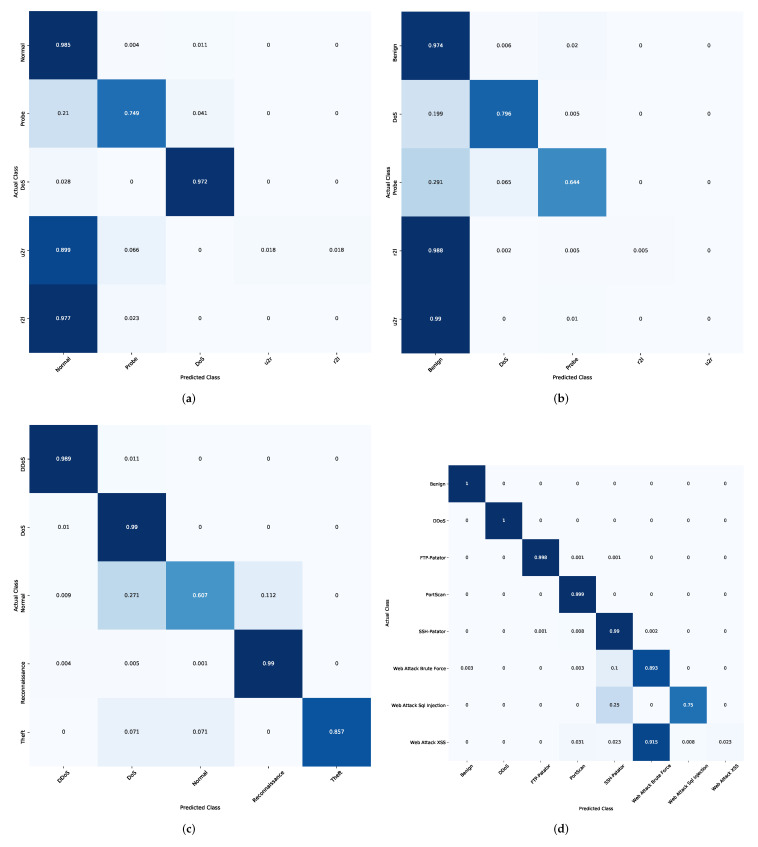
Confusion Matrix of developed method. (**a**) KDDCup99, (**b**) NSL-KDD, (**c**) BoT-IoT, (**d**) CICIDS-2017.

In addition, the results of the competitive algorithms in case of CICIDS-2017 dataset are given in Table 2 and Table 3. It can be observed that the proposed AQU obtained the best results, especially in the multi-classification. Moreover, by comparing the results of AQU with the other model in FS case, it can be noticed that its PIR of accuracy variant from 0.260 to 0.590. However, the PIR of recall, Precision, and F1-Measure is 0.210 to 0.590, 0.212 to 0.580, and 0.210 to 0.570. The same observation can be reached from Figure 7g,h that illustrate the PIR for each algorithm using CICIDS-2017 dataset. Figure 9 depicts the confusion matrix of developed method over the tested datasets.

The Friedman test [62] is used to assess if there are significant differences between the presented technique and others to further analyze the results. There are two hypotheses in this test: the first, known as the null hypothesis, supposes that there are no differences between the compared algorithms and is accepted the case of the *p*-value ≥ 0.05. Otherwise, the alternative hypothesis (second one) is adopted which assume a considerable difference in techniques. In the two cases, Table 4 displays the mean rank of each algorithm for the four datasets (i.e., binary and multi-classifications). The proposed AQU obtained the highest mean rank for all applied performance indicators in both scenarios of multi-classification, as can be seen from the results. There is also a substantial distinction between AQU and other approaches.

## 6. Conclusions

In this paper, a new approach was proposed for the internet of things (IoT) intrusion detection system (IDS). We leveraged the advances of swarm intelligence (SI) and deep learning techniques. The proposed approach works as follows. First, a designed conventional neural network (CNN) based feature extraction method was applied to obtain the related features from the input datasets. Second, a new variant of the recently developed Aquila optimizer (AQU) was used to select appropriate features and to reduce data dimensionality. The main idea of the developed AQU is to use its binary version to overcome the limitations of the traditional AQU algorithm. To evaluate the developed approach, we used four well-known public datasets, namely, CIC2017, NSL-KDD, BoT-IoT, and KDD99. Moreover, extensive comparisons were carried out with several optimization algorithms, such as WOA, BAT, TSO, GWO, FFA, MVO, and MFO, using several evaluation measures, such as precision, recall, and F1-Measure. The outcomes have confirmed the superiority of the developed AQU against all compared methods. There are still some limitations in the developed method, such as AQU, which can be addressed in future work. Moreover, different swarm intelligence methods will be considered with different deep learning architectures for IDS in the IoT environment.

## Figures and Tables

**Table 1:** The basic formulation of the confusion matrix, where TP represents true positive, FN indicates false negative, false positive is represented by FP, and TN represents true negative.

	Predicted Label	
**Actual Label**	**Positive**	**Negative**
**Postive**	TP	FN
**Negative**	FP	TN

**Table 2:** Results of developed AQUa for the datasets in case of multi-classification.

		Training				Testing			
		${\mathrm{AV}}_{\mathrm{Acc}}$	${\mathrm{AV}}_{\mathrm{Sens}}$	${\mathrm{AV}}_{\mathrm{Prec}}$	**F1**	${\mathrm{AV}}_{\mathrm{Acc}}$	${\mathrm{AV}}_{\mathrm{Sens}}$	${\mathrm{AV}}_{\mathrm{Prec}}$	**F1**
KDD99	PSO	90.447	93.458	90.358	90.358	82.783	85.793	84.640	83.109
	WOA	92.275	93.126	92.414	97.304	84.375	85.225	82.501	87.351
	BAT	98.007	98.247	94.847	97.337	90.347	90.587	89.134	**90.093**
	TSO	95.439	94.919	91.027	97.437	87.536	87.016	80.791	87.479
	GWO	95.513	92.383	94.062	98.482	87.618	84.488	84.131	88.533
	FFA	91.988	93.368	97.328	91.538	84.318	85.698	**91.609**	84.285
	MVO	99.515	92.835	96.483	94.433	91.615	84.935	86.649	84.480
	MFO	96.073	97.123	**97.631**	98.371	88.175	89.225	87.763	88.420
	AQU	**99.920**	**99.917**	97.542	**99.920**	**99.919**	**92.042**	89.824	89.987
BIoT	PSO	**99.483**	**99.483**	**99.483**	99.483	98.942	98.972	98.941	98.940
	WOA	99.472	99.472	99.472	99.472	98.956	98.964	98.957	99.005
	BAT	99.475	99.475	99.475	99.474	99.019	**99.021**	98.987	99.012
	TSO	99.460	99.460	99.459	99.459	98.986	98.981	98.941	99.005
	GWO	99.477	99.477	99.476	99.476	98.990	98.959	98.975	99.019
	FFA	99.479	99.479	99.478	99.478	98.954	98.968	99.007	98.949
	MVO	99.468	99.468	99.468	99.468	**99.031**	98.964	99.000	98.980
	MFO	99.480	99.480	99.480	99.480	98.998	99.009	**99.013**	**99.020**
	AQU	98.925	98.925	98.904	98.925	98.926	98.904	98.905	98.904
NSL-KDD	PSO	90.118	93.128	90.020	90.019	66.092	69.102	68.913	61.940
	WOA	91.947	92.797	92.080	96.968	67.951	68.801	71.131	68.907
	BAT	97.669	97.909	94.501	96.989	73.671	73.911	73.501	68.905
	TSO	95.078	94.558	90.657	97.067	71.330	70.810	71.298	69.697
	GWO	95.182	92.052	93.724	98.143	71.066	67.936	72.151	69.948
	FFA	91.660	93.040	96.991	91.201	67.437	68.817	75.873	62.944
	MVO	99.182	92.502	96.145	94.093	75.224	68.544	75.200	66.098
	MFO	95.745	96.795	97.297	98.035	71.626	72.676	76.122	69.844
	AQU	**99.344**	**99.344**	**99.298**	**99.315**	**76.002**	**76.002**	**81.719**	**71.602**
CIC2017	PSO	99.650	99.370	99.590	99.750	99.380	99.100	99.320	99.480
	WOA	99.690	99.690	99.490	99.450	99.430	99.430	99.240	99.190
	BAT	99.490	99.640	99.630	99.440	99.230	99.380	99.360	99.180
	TSO	99.680	99.710	99.750	99.680	99.420	99.450	99.480	99.420
	GWO	99.370	99.560	99.430	99.380	99.110	99.300	99.180	99.120
	FFA	99.450	99.740	99.480	99.600	99.200	99.490	99.220	99.350
	MVO	99.530	99.370	99.390	99.410	99.270	99.110	99.120	99.150
	MFO	99.360	99.430	99.370	99.480	99.100	99.170	99.120	99.220
	AQU	**99.911**	**99.909**	**99.889**	**99.910**	**99.911**	**99.910**	**99.910**	**99.888**

**Table 3:** Results of developed AQUa for the datasets in case of Binary.

		Training				Testing			
		${\mathrm{AV}}_{\mathrm{Acc}}$	${\mathrm{AV}}_{\mathrm{Sens}}$	${\mathrm{AV}}_{\mathrm{Prec}}$	**F1**	${\mathrm{AV}}_{\mathrm{Acc}}$	${\mathrm{AV}}_{\mathrm{Sens}}$	${\mathrm{AV}}_{\mathrm{Prec}}$	**F1**
KDD99	PSO	90.449	93.459	90.359	90.359	82.775	85.785	84.638	92.702
	WOA	92.278	93.128	92.418	97.308	84.608	85.458	86.699	92.705
	BAT	94.992	98.662	92.922	91.782	87.384	91.055	87.280	**92.751**
	TSO	95.298	94.592	90.825	97.332	87.593	87.090	85.280	92.541
	GWO	95.518	92.388	94.068	98.488	87.860	84.730	88.357	92.716
	FFA	91.987	93.367	97.327	91.537	84.327	85.707	91.614	92.713
	MVO	99.519	92.839	96.489	94.439	91.844	85.164	90.765	92.701
	MFO	96.079	97.129	**97.639**	98.379	88.413	89.463	91.922	92.710
	AQU	**99.922**	**99.922**	92.256	**99.922**	**99.922**	**92.256**	**94.283**	92.683
BIoT	PSO	99.899	99.929	99.898	99.898	99.898	99.928	99.896	99.896
	WOA	99.918	99.926	99.919	99.967	99.916	99.924	99.916	99.965
	BAT	99.975	99.977	99.943	99.968	99.973	99.975	99.941	99.966
	TSO	99.949	99.944	99.905	99.969	99.947	99.942	99.903	99.967
	GWO	99.950	99.919	99.935	99.979	99.948	99.917	99.933	99.977
	FFA	99.915	99.928	99.968	99.910	99.913	99.927	99.966	99.908
	MVO	99.990	99.923	99.959	99.939	99.989	99.922	99.958	99.937
	MFO	99.956	99.966	99.971	99.978	99.954	99.964	99.969	99.976
	AQU	**99.995**	**99.994**	**99.993**	**99.995**	**99.994**	**99.993**	**99.992**	**99.992**
NSL-KDD	PSO	90.133	93.143	90.043	90.043	67.575	70.585	73.882	67.163
	WOA	91.959	92.809	92.099	96.989	69.409	70.259	75.972	74.115
	BAT	97.693	97.933	94.533	97.023	75.192	75.432	78.473	74.197
	TSO	95.091	94.571	90.681	97.091	72.078	71.558	73.656	73.786
	GWO	95.202	92.072	93.753	98.172	72.944	69.814	77.801	75.609
	FFA	91.673	93.053	97.013	91.223	69.218	70.598	80.944	68.451
	MVO	99.197	92.517	96.167	94.117	76.466	69.786	79.835	71.059
	MFO	95.760	96.810	97.320	98.060	73.187	74.237	81.176	75.162
	AQU	**99.348**	**99.348**	**99.350**	**99.348**	**77.382**	**77.382**	**83.692**	**77.077**
CIC2017	PSO	99.687	99.407	99.627	99.387	99.687	99.407	99.627	99.787
	WOA	99.730	99.531	99.537	99.470	99.737	99.737	99.537	99.497
	BAT	99.537	99.647	99.667	99.472	99.537	99.687	99.667	99.487
	TSO	99.724	99.654	99.744	99.436	99.725	99.755	99.785	99.725
	GWO	99.417	99.607	99.477	99.427	99.417	99.607	99.477	99.427
	FFA	99.497	99.601	99.517	99.470	99.497	99.787	99.517	99.647
	MVO	99.577	99.417	99.427	99.457	99.577	99.417	99.427	99.457
	MFO	99.407	99.477	99.417	99.427	99.407	99.477	99.417	99.527
	AQU	**99.996**	**99.996**	**99.996**	**99.996**	**99.997**	**99.997**	**99.997**	**99.997**

**Table 4:** Results of algorithms using Friedman test.

	PSO	MVO	GWO	MFO	WOA	FFA	BAT	AQU	TSO
	Binary classification								
Accuracy	1	8	5.33	6.33	3	2	6	9	4.33
Recall	4.66	1.66	1.33	7	3	4.33	8	9	6
Precision	1.33	6	4.33	8	3	7	4.66	9	1.66
F1-Measure	1.66	2.66	7.66	6.33	4.33	3.33	6.33	9	3.66
	Multi classification								
Accuracy	1	8	4.66	6	3	2	7	9	4.33
Recall	5	2.16	1	7	2.83	4	8	9	6
Precision	2.16	5.66	3.66	7.33	2.33	7.66	5.66	8.66	1.83
F1-Measure	1	3	7.33	7	4.33	2	6.5	8.66	5.16

## Data Availability

Data used in this study are public datasets as mentioned in the main text.
